# Best diagnostic approach for the genetic evaluation of fetuses after intrauterine death in first, second or third trimester: QF-PCR, karyotyping and/or genome wide SNP array analysis

**DOI:** 10.1186/1755-8166-7-6

**Published:** 2014-01-16

**Authors:** Angelique JA Kooper, Brigitte HW Faas, Ilse Feenstra, Nicole de Leeuw, Dominique FCM Smeets

**Affiliations:** 1 Department of Human Genetics, Radboud university medical center, Nijmegen, The Netherlands

**Keywords:** Intrauterine fetal death, IUFD, DNA, QF-PCR, Karyotyping, SNP array

## Abstract

**Background:**

The aim of this study was to evaluate the best diagnostic approach for the genetic analysis of samples from first, second and third trimester intrauterine fetal deaths (IUFDs). We examined a total of 417 IUFD samples from fetuses with and without congenital anomalies. On 414 samples, karyotyping (N = 46) and/or rapid aneuploidy testing by QF-PCR (N = 371) was performed). One hundred sixty eight samples with a normal test result were subsequently tested by genome wide Single Nucleotide Polymorphism (SNP) array analysis. Three samples were only analyzed by array.

**Results:**

In 50 (12.0%) samples an aneuploidy was detected by QF-PCR and/or karyotyping, representing 47.1% of first, 13.2% of second and 3.4% of third trimester pregnancies. Karyotyping and QF-PCR failed in 4 (8.7%) and 7 (1.9%) samples, respectively, concerning mostly contaminated amniotic fluid samples from third trimester pregnancies.

Clinically relevant aberrations were identified in 4.2% (all fetuses with malformations) of the 168 samples tested by SNP array. Inherited copy number variants (CNVs) were detected in 5.4% and 8.9% showed CNVs of unknown clinical relevance as parental inheritance could not be studied yet. In a sample from a fetus suspect for Meckel-Grüber syndrome, the genotype information from the SNP array revealed various stretches of homozygosity, including one stretch encompassing the *CEP290* gene. Subsequent *CEP290* mutation analysis revealed a homozygous, pathogenic mutation in this gene.

**Conclusions:**

Based on our experience we recommend QF-PCR as the first-line test in IUFD samples of first and second trimester pregnancies to exclude aneuploidy before performing array analysis. The chance to detect aneuploidy in third trimester pregnancies is relatively low and therefore array analysis can be performed as a first-tier test. A tissue sample, instead of amniotic fluid, is preferred because of a higher success rate in testing.

We emphasize the need for analysis of parental samples whenever a rare, unique CNV is detected to allow for better interpretation of such findings and to improve future pregnancy management. Furthermore, we illustrate the strength of SNP arrays for genotype analysis, even though we realize it is crucial to have detailed phenotypic information to make optimal use of the genotype data in finding candidate recessive genes that may be related to the fetal phenotype.

## Background

There are numerous causes of fetal death, including genetic conditions, infections, placental abnormalities, and fetal-maternal hemorrhage. Valuable tests for the evaluation of fetal death are perinatal autopsy, placental examination, cytogenetic analysis, and testing for fetal maternal hemorrhage [1,2]. The first trimester of pregnancy, usually defined as the period from fertilization until the 13th week of gestation, is the most sensitive time of development for the conceptus and a relatively high incidence of first trimester spontaneous abortion is reported. Although maternal exposure to certain teratogens and possible immune rejection of the conceptus do occur, the most common cause of first trimester spontaneous abortion is a chromosomal abnormality [3-8]. Chromosomal abnormalities found in second trimester losses are similar to those found in live births; the most common are trisomies 13, 18, and 21, monosomy X, and sex chromosome polysomies [9]. Second (13–27 weeks) and third trimester (28–42 weeks) fetal death can also be attributed to many other single or multiple causes. Beside fetal anomalies placentation and cord morbidities, or maternal co-morbidities may play a major role. However, a cause-and-effect relationship is often difficult to establish. The most common cause of intrauterine fetal death (IUFD) in the third trimester appears to be umbilical cord accidents [4-6].

Karyotyping of IUFD samples requires viable cells. As karyotyping fails in up to 40% of the cases due to culturing failure, molecular testing of IUFD samples by Multiplex Ligation-dependent Probe Amplification (MLPA) or Quantitative Fluorescent Polymerase Chain Reaction (QF-PCR), for the detection of the most common aneuploidies, has proven to be very helpful as cell culture is not required [10,11]. The microarray technology already proved its value in post- and prenatal diagnostics as it enables the detection of submicroscopic aberrations (gains and losses) with a very high resolution [12-20]. Moreover, it overcomes many of the limitations of routine karyotyping [7,10,21,22]. Since January 2010 our diagnostic centre routinely performs array analysis in IUFD samples associated with congenital anomalies after exclusion of the most common aneuploidies. Since February 2012 array analysis is also been performed for IUFD samples without congenital anomalies (and without a common aneuploidy).

In this retrospective study, we evaluated the results of various genetic tests in samples of IUFD from first, second and third trimester pregnancies. Next to the results of traditional karyotyping and QF-PCR, we illustrate the strength of Single Nucleotide Polymorphisms (SNP) array analysis based on genotyping data.

## Methods

From January 2010 until August 2012, 417 samples of fetal death *in utero* were investigated. Genetic studies were performed on all IUFD samples either by analysis of material obtained after invasive prenatal testing before induction of labor (amniotic fluid or chorionic villi, N = 57) or by analysis of postpartum obtained tissue of the fetus (fetal skin, placental and/or umbilical cord material, N = 360). All samples were analyzed at the Radboud university medical center in Nijmegen for genetic examination. Gestational age of the pregnancy and clinical information about the fetus were collected from the genetic test request form.

### DNA for QF-PCR

Tissue samples were minced and treated with collagenase to obtain a cell suspension. Subsequently, DNA was isolated following standard procedures by robot (Chemagic Magnetic Separation Module 1 from Chemagen, Baesweiler, Germany).

From chorionic villi, the cytotrophoblast and mesenchymal core fraction were enzymatically dissociated with trypsin/EDTA followed by collagenase treatment. Genomic DNA for QF-PCR was extracted from both villi fractions. DNA was extracted from 1 ml uncultured amniotic fluid using a Chelex based procedure (Instagene Matrix, Bio-Rad Laboratories, Hercules, CA, USA).

### DNA for array

DNA isolated from tissue samples for QF-PCR was also used for array.

From chorionic villi, only DNA isolation from the mesenchymal core fraction was performed for array analysis using the QIAamp DNA Mini Kit (Qiagen Benelux BV, Venlo, the Netherlands), following the instructions of the manufacturer.

From uncultured amniotic fluid, DNA from 6 ml was isolated, using the QIAamp MinElute Virus spin kit (Qiagen Benelux BV, Venlo, the Netherlands) and eluted in 50 μl of elution buffer.

### QF-PCR

Two different QF-PCR kits were used: tissue samples were tested for aneuploidies of the chromosomes 13, 15, 16, 18, 21, 22, X, or Y (kit Devyser Extend, Cytogen, Sinn, Germany), while chorionic villi and amniotic fluid samples were tested for aneuploidies of the chromosomes 13, 18, 21, X and Y (Aneufast, Genomed Ltd, UK).

Whenever an aneuploidy was detected, cytogenetic evaluation of parental blood followed to study whether the fetal abnormality could be due to a parental rearrangement.

### Karyotyping

In 46 of the 57 chorionic villi or amniotic fluid samples, karyotyping was the first-line test due to the fact that at that time the QF-PCR was not yet implemented as a first-line test for prenatal IUFD samples. Karyotyping was performed following standard procedures.

In three samples, culturing of the fetal material and/or QF-PCR could not be carried out and, therefore, only array analysis was performed.

### Array

After a normal QF-PCR or karyotype result, genome wide array analysis was performed on DNA from 168 IUFD samples from fetuses with or without malformations (before February 2012 array was only performed on IUFD samples with malformations or on request, from February 2012 routinely on all IUFD samples).

Seventy one samples were tested using the Affymetrix GeneChip 250 k (NspI) SNP array platform (Affymetrix, Inc, Santa Clara, California, USA), which contains 25-mer oligonucleotides representing a total of 262,264 SNPs (Method, see [13]). Data were analyzed with the CNAG software package [14]. The other 97 array analyses were carried out following the manufacturer’s protocols on the high resolution CytoScan HD array platform which contains more than 2.6 million markers, including 750,000 genotype-able SNPs and 1.9 million non-polymorphic probes. Data were analyzed with the Affymetrix Chromosome Analysis Suite (ChAS) software (Affymetrix, Inc, Santa Clara, California, USA). The two major quality control metrics for Affymetrix array are the Median Absolute Pairwise Difference (MAPD) score which applies to copy number probes and the SNP-QC which applies to SNP probes. In our diagnostic setting, the values for these parameters need to be **≤** 0.25 for MAPD and ≥ 0.15 for SNP-QC.

We set the cut-offs for our detection criteria for copy number variants (CNVs) at 20 kb for gains, 10 kb for losses and 1,250 kb for Regions Of Homozygosity (ROH). Our reporting criteria for CNVs depend on the gene content and on the size. Follow-up testing on parental samples is, based on the size of the CNV, performed by array, Fluorescence In Situ Hybridization (FISH) and/or karyotyping. An overview of our recommendations concerning the detection and reporting criteria for diagnostic array results and follow-up testing is shown in Table 1. The breakpoint positions of each aberrant region were converted to UCSC hg19 (UCSC Genome Browser, release February 2009).

**Table 1:** Recommendations concerning the detection and reporting criteria for diagnostic array results and follow-up testing

**Detection criteria**	**Gain**	**Loss**	**ROH**	
Minimum marker count	10	10	500 (SNP probes)	
Minimum size (kb)	20	10	1250	
**Reporting criteria**	**Gene content of CNV**	**Size of CNV (kb)**		
	Known disease gene, matching the phenotype	<20		
	Known disease gene(s)*	>20		
	No disease gene(s)	>100		
	No genes	>500		
	**ROH**	**Size of ROH (Mb)**		
		> 10	Genomic Oligoarray and SNP array evaluation tool v2.0 ^[24]^ **→** recessive disease causing genes **→** mutation detection	Positive → Parental mutation carrier analysis
		< 10	In case of a specific clinical suspicion for a known syndrome **→** Genomic Oligoarray and SNP array evaluation tool v2.0 ^[24]^ →recessive disease causing genes **→** mutation detection	Positive **→** Parental mutation carrier analysis
**Parental testing**	**Type of CNV**	**Follow-up test**		
	Uncertain	Array analysis		
	Clinically relevant:			
	- cytogenetically visible (> 5–10 Mb)	Karyotyping		

	- submicroscopic aberration (<1-10 Mb)	FISH		

*Depending on the referral reason for array, in this case intrauterine fetal death, a certain CNV will sometimes not be reported, even though our general reporting criteria are met. For example, if it concerns a 50 kb loss in an OMIM disease gene that is not involved in embryonic and fetal development. Alternatively, a certain CNV is reported, but in the explanatory text of the array report it is mentioned that this CNV is not very likely causative for the IUFD.

CNVs were classified as (a) benign, (b) likely/probably benign, (c) of uncertain clinical relevance, (d) of unknown clinical significance because of unknown inheritance, (e) likely/possibly clinically relevant or (f) clinically relevant.

A variant was categorized as benign if its full length had been reported in at least three apparently unaffected individuals as listed in the Database of Genomic Variants [23] or our in-house databases containing array data from healthy control individuals (i.e., volunteers, blood donors, etc.). A CNV was likely/probably benign when an identical CNV was inherited from an healthy parent and likely/probably clinically relevant whenever the CNV had been described in a single case report but with well-defined phenotype, specific and relevant to the IUFD. Clinically relevant variants had evidence of pathogenicity according to the published literature, often containing a gene known to be relevant in fetal development or fetal death as listed in the Online Mendelian Inheritance in Man (OMIM) database. Variants that did not meet the criteria for classification as clinically relevant or benign were classified as CNVs of uncertain clinical significance, or as CNVs of unknown clinical significance because of unknown inheritance because parental inheritance could not be determined yet.

In addition to copy number analysis, SNP array data analysis also enabled genotyping of the array data and the detection of homozygous stretches. Regions Of Homozygosity (ROH) with a size > 10 Mb were evaluated for autosomal recessive conditions in fetuses with a clinical phenotype using a clinical evaluation tool for SNP arrays (Genomic Oligoarray and SNP array evaluation tool v2.0) [24]. This tool systematically searches through relevant databases including the OMIM database, the University of California at Santa Cruz Genome Browser (UCSC), and the National Center for Biotechnology Information (NCBI) database, to rapidly identify disease genes mapping to the ROH to enumerate associated autosomal recessive clinical disorders and their clinical features.

## Results

**Figure 1:**
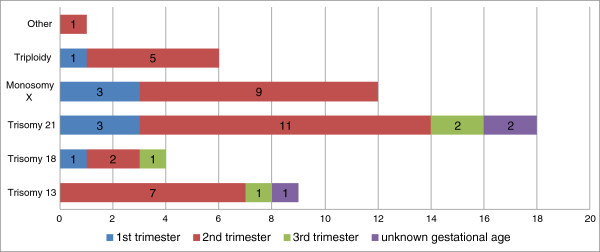
Distribution, number and type of aneuploidy detected in the cohort of 417 IUFD samples.

An overview of all samples and diagnostic test results is shown in Figure 1.

### QF-PCR/Karyotyping

**Figure 2:**
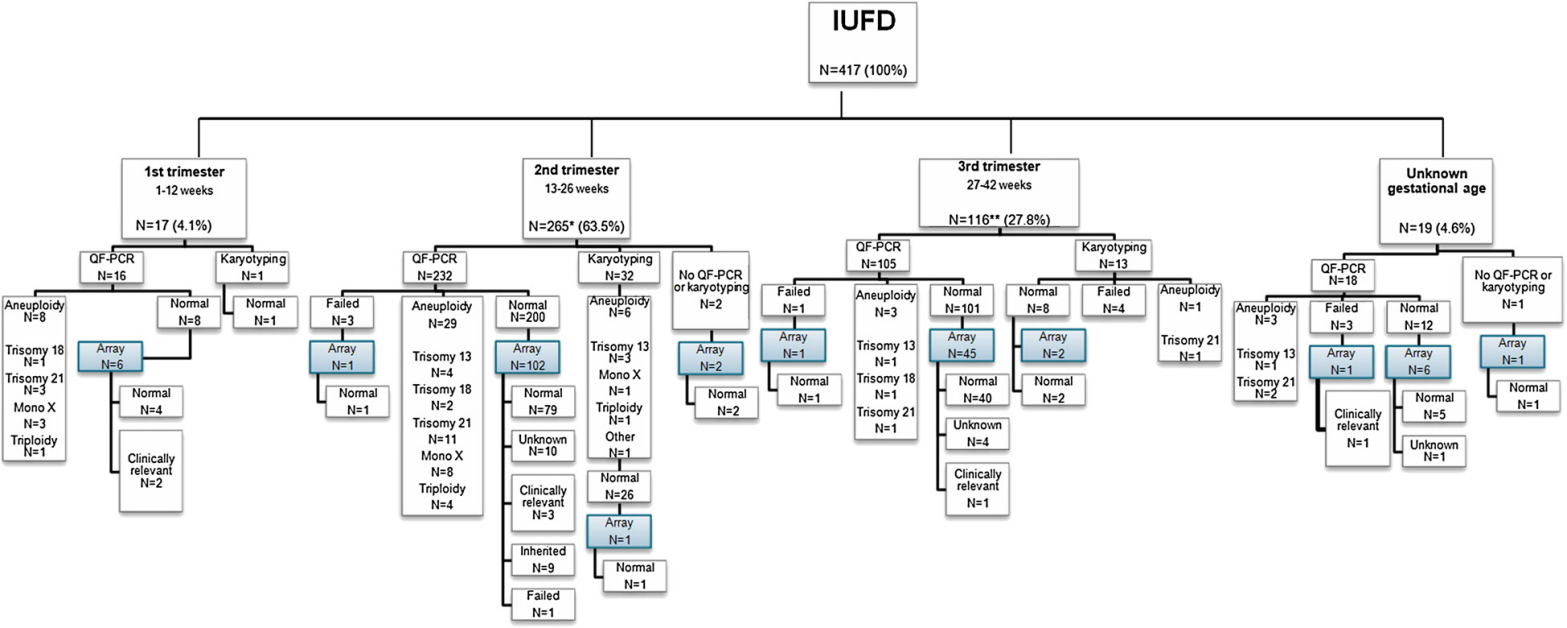
**Overview of diagnostic testing in 417 IUFD samples.** Aneuploidy testing (by QF-PCR or karyotyping) is categorized as normal, aneuploidy, not performed or failed. Microarray results are indicated as normal (benign CNV), clinically relevant, unknown (CNV of unknown inheritance), inherited (likely/probably benign CNV) or failed. Mono X: monosomy X; *sample tested by QF-PCR and karyotyping; **two samples tested by both QF-PCR and karyotyping.

In 50 (12.0%) samples an aneuploidy was detected. This was 47.1% (8/17) in first trimester samples, 13.2% (35/265) in second trimester samples, 3.4% (4/116) in third trimester samples, and 15.8% (3/19) in the samples of unknown gestational age. The aneuploidies included trisomy 13 (N = 9), trisomy 18 (N = 4), trisomy 21 (N = 18), monosomy X (N = 12), triploidy (N = 6) and one mosaic trisomy 7 (Figure 2).

Karyotyping (N = 46) failed in four (8.7%) amniotic fluid samples. These four samples were from third trimester pregnancies and were contaminated with old blood.

QF-PCR (N = 368) failed in seven samples (1.9%). Two of these were amniotic fluid samples which were contaminated with old blood. In both cases, diagnostic testing could successfully be performed by array. In the first case, also umbilical cord was received and examined, and in the second case amniotic fluid cells could be cultured and used for DNA isolation and array. The QF-PCR test of two skin biopsies failed because DNA was of poor quality due to degradation. We did not obtain interpretable results by QF-PCR in three placenta samples because of maternal cell contamination. In one sample with maternal contamination array was performed, the sample was from a male fetus with an paternally inherited unbalanced translocation t(17;18).

### Array

In 168 samples examined by array one test failure (Cytoscan HD) occurred because the DNA was of poor quality due to degradation. Six samples did not meet our quality criteria and were therefore analyzed with an adjusted resolution of 1 Mb.

#### Copy number variants (CNVs)

A normal array profile was detected in 136 out of 168 (81.0%) samples. In seven (4.2%) samples, one or more (likely) clinically relevant CNVs were detected (Table 1), with sizes ranging from 1.1 to 48.0 Mb. To define whether the CNVs had occurred *de novo*, were inherited, or caused by the presence of a parental balanced translocation, additional parental testing was performed in six of the seven samples. Only in case id 60 blood was requested but not yet received. Parental karyotyping was performed in five samples and normal karyotypes were obtained in nine parents. In case id 206, the father appeared to be carrier of a balanced reciprocal translocation 46,XY,t(17;18)(q23;q21.1), as is also indicated in the fetal karyotype in Table 2. Parental testing was performed by FISH in case id 296 and revealed a normal 22q11.2 FISH pattern in both parents.

**Table 2:** CNVs that are clinically relevant (N = 7)

**Case id**	**Pregnancy trimester**	**Clinical features**	**Array platform**	**Size (Mb)**	**Chromosomal region**	**Start-end Mb position (Hg19)**	**CNV**	**Disease-causing OMIM genes involved (UCSC feb 2009)**
**60**	first	Hydrops fetalis	CytoScan® HD	8.4	3p24.2p24.3	17269256-25630783	loss	*THRB*
**206**	unknown	MCA	250 k	8.4	17q23.1q25.3	70175362-78598059	gain	~ 19 genes*
				26.8	18q21.2q23	49229300-76115293	loss	~ 13 genes*
							t(17;18)pat	
**212**	third	Unilateral talipes calcaneovalgus, unilateral ear tag	250 k	11.8	5p15.2p15.33	81949-11834131	dn loss	*SDHA, SCL6A19, TERT, SLC6A3, NDUFS6, NSUN2, MTRR, CCT5*
				4.3	5p15.1p15.2	11857228-16124168	dn gain	*DNAH5, ANKH*
**274**	second	Edema (hands and feet)	CytoScan® HD	48.0	1q32.1q44	201214014-249224685	dn gain	~50 genes*
				1.1	9p24.3	203862-1293115	dn loss	*DOCK8, KANK1*
				37.5	9p13.1p24.3	1293354-38787480	dn gain	~30 genes*
**296**	second	Hypertelorism, micrognathia, microencephaly, flat face	CytoScan® HD	3.2	22q11.21	18644791-21800798	dn loss	*PRODH, GP1BB, TBX1, COMT, RTN4R, SCARF2, HCF2, SNAP29*
**309**	first	Exencephaly	CytoScan® HD	18.4	18p11.32p11.1	136227-18521286	dn loss	*SMCHD1, LPIN2, TGIF, NDUFV2, APCDD1, GNAL, AFG3L2, MC2R*
				19.3	18q21.32q23	58738938-78014124	dn gain	*PIGN, TNFRSF11A, BLC2, RTNN, CYB5A, TSHZ1, CTDP1*
**317**	first	Hydrops fetalis	CytoScan® HD	23.0	9q22.33q33.2	101052575-124018186	dn loss	~20 genes*

*Number of disease-causing OMIM genes > 10 → genes not specified.

In fifteen (8.9%) samples CNVs (12/15 with sizes <1 Mb, 3/15 with sizes between 1–1.4 Mb) were detected of unknown inheritance (see Table 3). Although parental samples were requested for carrier testing, these have not been received so far.

**Table 3:** CNVs of unknown clinical relevance (due to unknown parental inheritance)

**Case id**	**Pregnancy trimester**	**Clinical features**	**Array platform**	**Size (kb)**	**Chrom region**	**Start-end Mb position (Hg19)**	**C CNV**	**Ref Seq (and/or disease-causing OMIM) genes involved (UCSC feb 2009)**
**2**	second	Suspect twin to twin transfusion syndrome (TTTS), clenched fist both hands, monozygotic twin	250 k	663	4q34.1	173762947-174426181	loss	*GALNTL6, GALNT7, HMGB2, SAP30, SCRG1*
**7**	second	IUGR	250 k	447	3q21.2	125498921-125945498	gain	*MIR548I1, FAM86JP, ALG1L, ROPN1B, SLC411A3, ALDH1L1, ALDH1L1-AS1 and AS2*
**12**	second	MCA	250 k	587	2p12	77036873-77624262	loss	*LRRTM4*
**14**	second	Unknown, recurrent IUFD	250 k	1,200	6q16.1	95641761-96851236	gain	*MANEA, FUT9, MANEA-AS1*
**16**	second	Hygroma colli	250 k	1,400	1q21.1	143570846-144929606	loss	>10 RefSeq genes*, mutation analysis for Noonan genes: *NRAS, SHOC2, GBL, RAF1, SOS1, KRAS, PTPN11* negative
**21**	third	Hydrocephaly	CytoScan® HD	383	16p13.3	3945203-4328143	loss	*ADCY9, SRL, LOC100507501,TFAP4* (proximal to OMIM gene *CREBBP*)
**30**	second	Potter’s sequence, renal agenesis, facial dysmorfisms, single palmar crease	250 k	1,005	1p32.3	53484565-54489583	gain	>10 RefSeq genes* and 3 disease-causing OMIM genes: *SCP2, CPT2, LRP8*
**34**	unknown	unknown	CytoScan® HD	830	3p24.1	26641410-27471832	gain	*LRRC3B, NEK10, SLC4A7*
**39**	second	None	CytoScan® HD	828	Xq28	148839499-149667835	gain	*MAGEA9B, MAGEA9, MAGEA8-AS1, MAGEA8, CXorf40B, LINC00894, MIR2114* and 1 disease-causing OMIM gene: *MAMLD1*
**47**	second	None	CytoScan® HD	358	18q21.2	52900743-53258705	gain	*MIR4529* and disease-causing OMIM gene *TCF4*. Duplication *TCF4* confirmed by MLPA.
**50**	third	None	CytoScan® HD	545	8p21.3p22	18825888-19370744	loss	*FSD3, LOC100128993, SH2D4A, CSGALNACT1*
**20**	third	None	CytoScan® HD	490	3q29	196592132-197081797	gain	*SENP5, NCBP2, NCBP2-AS2, PIG2, MFI2, MFI2-AS1, DLG1, DLG1-AS1, MIR4797*
**55**	second	None	CytoScan® HD	611	16p11.2	29567296-30177917	loss	>10 RefSeq genes* and 3 disease-causing OMIM genes: *KIF22, PRRT2, ALDOA*
**56**	second	Unknown	CytoScan® HD	767	22q11.21	21033398-21800798	loss	>10 RefSeq genes* and 2 disease-causing OMIM genes: *HCF2, SNPA29*
**59**	third	None	CytoScan® HD	266	8p23.3	158049-423802	loss	*RPL23AP53, ZNT596, FAM87A, FBX025*

*Number of Ref Seq genes > 10 → genes not specified.

In nine (5.4%) samples a parentally inherited CNV was detected (Table 4): these were all gains ranging with sizes < 1 Mb in 8/9 cases and 1.2 Mb in 1/9 cases (6 out of 8 being maternally inherited).

**Table 4:** Inherited CNVs (N = 9)

**Case id**	**Pregnancy trimester**	**Clinical features**	**Array platform**	**Size (kb)**	**Chromosomal region**	**Inheritance**	**Start-end Mb position (Hg19)**	**CNV**
**242**	second	Recurrent miscarriages, micrognatia	250 k	559	22q12.3	maternal	31829608-32388222	gain
**257**	second	Unknown	250 k	400	1p31.1	maternal	71586877-71986553	gain
**272**	second	Hydrops fetalis, low-set ears, Pierre Robin Sequence (PRS)	CytoScan® HD	303	Xq24	maternal	118733984-119037053	gain
**280**	second	Unknown	CytoScan® HD	251	6q27	paternal	169570833-169822659	gain
**281**	second	Mild facial dysmorfic features, hypertelorism, long philtrum	CytoScan® HD	1,200	1q43	maternal	236830156-238009186	gain
**289**	second	Agenesis of the corpus callosum (ACC), hydrops fetalis, ascites, mild ventriculomegaly	CytoScan® HD	260	9q21.11	maternal	71570080-71842392	gain
**316**	second	Unknown	CytoScan® HD	367	16p13.3	maternal	5393095-5760407	gain
**359**	second	None (recurrent miscarriages)	CytoScan® HD	990	7q11.21	paternal	65329349-66417018	gain
**65**	second	Unknown (macerated fetus)	CytoScan® HD	294	Xp22.31	paternal	8439472-8733564	gain

**Figure 3:**
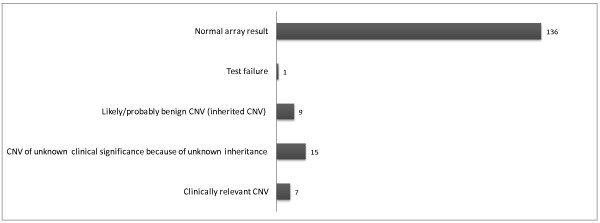
Overview of array results: type and number of CNVs detected in 168 samples with IUFD.

An overview of all array results is shown in Figure 3.

#### Regions of Homozygozity (ROH)

**Figure 4:**
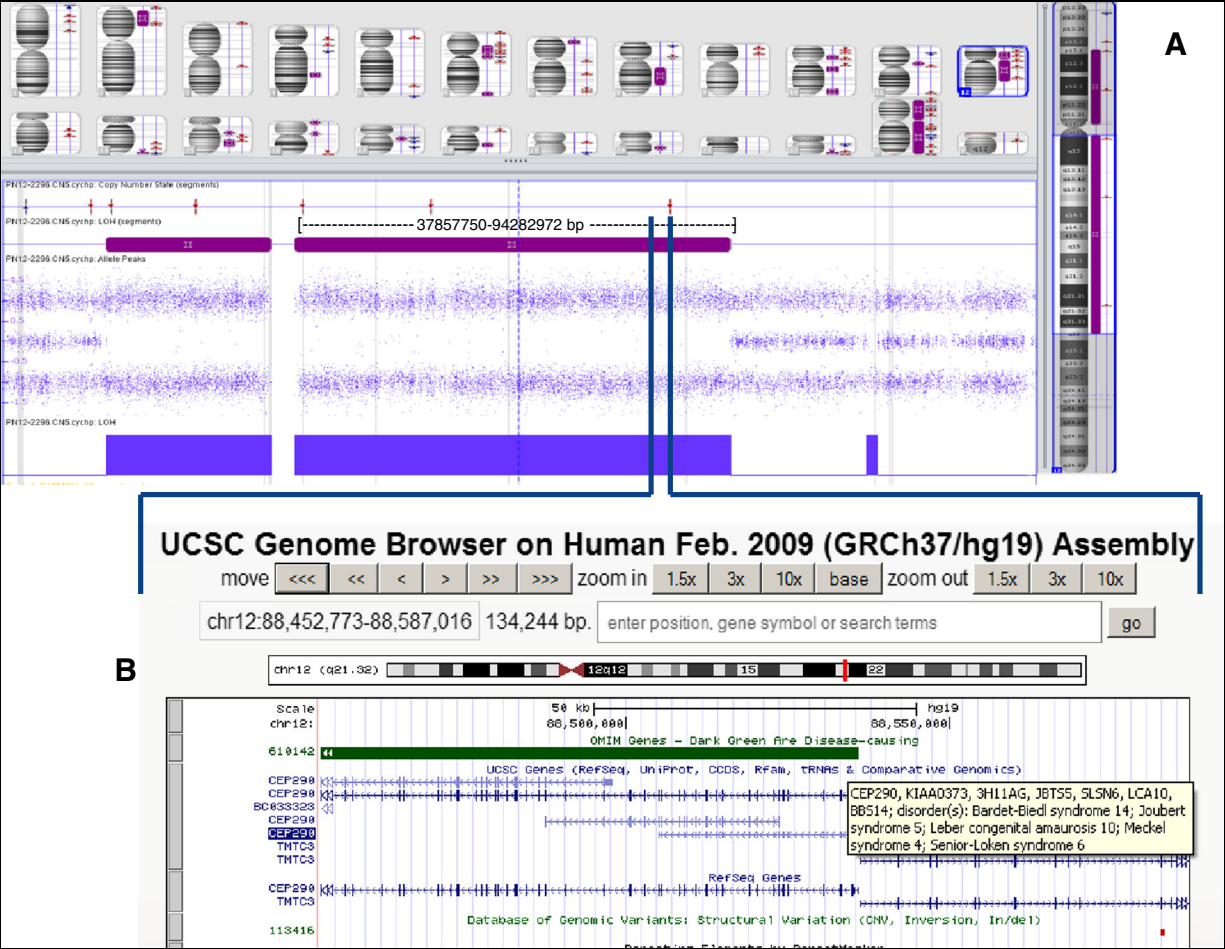
**A region of homozygosity in chromosome 12, including the *CEP290 *** **gene.**

In 25 samples, one or more autosomal ROHs were detected with a size ≥ 5 Mb. From 11 of these samples fetal clinical features were known. By using the online SNP array evaluation tool [24], all genes with recessive inheritance were enumerated to map with the clinical features. In one of these samples (case id. 336), a homozygous mutation in a recessive disease gene was detected. Case id. 336 is a cord biopsy sample from a fetus, from consanguineous parents, that died at 14 weeks of gestation. Postpartum clinical features mentioned on the request form were meningo-encephalocele, a single umbilical artery and suspect for Meckel-Grüber syndrome. Meckel-Grüber syndrome is an autosomal recessive, early embryonic multi-systemic disorder (MKS: OMIM 249000). So far, nine different loci have been mapped, including the *CEP290* gene. DNA isolated from a cord biopsy showed a normal male profile with QF-PCR and array. However, 12.5% homozygosity of the autosomal genome was detected, including a pericentromeric segment of ~80 Mb on chromosome 12 encompassing >50 genes, among which the *CEP290* gene (Figure 4). Subsequent mutation analysis of this gene led to the identification of a pathogenic homozygous missense mutation c.3418G > T. Both parents appeared to be heterozygous carrier of this mutation.

## Discussion and conclusions

In the present study, we evaluated different genetic tests (QF-PCR, karyotyping and array analysis) in the examination of IUFD samples. Our results show that a common aneuploidy was detected in 12% of the samples which is comparable to a previous report of Korteweg et al. [2] who showed 13% chromosomal abnormalities in a heterogeneous cohort of fetal deaths. The percentage of aneuploidies was highest (47%) in first trimester pregnancies. This is in line with the reported incidence of 30-60% of chromosomal anomalies causing early fetal demise [24-28]. Although cost aspects were not evaluated in our study, aneuploidy detection is cheaper by QF-PCR than by array and therefore QF-PCR analysis is the preferred first tier technique before array analysis to exclude the most common aneuploidies, in particular in case of a first and second trimester IUFD.

The highest incidence of test failures occurred in amniotic fluid samples because of sample contamination. In 2011, we therefore changed our strategy and asked gynecologists to send a fetal biopsy in cases of IUFD instead of amniotic fluid. The quantity of DNA obtained from fetal tissue is usually sufficient, it is clean and therefore suitable for testing by both QF-PCR and array analysis and additional sequencing of single genes in case of a suspected monogenic disorder.

In our cohort with a normal QF-PCR result, a clinically relevant CNV was detected by array in 4.2% of the samples, varying in size from 1.1 to 48.0 Mb and all concerning fetuses with malformations. This is lower than reported in literature. However, in these papers, the genome-wide prevalence of CNVs was examined in truly unexplained stillbirth, resulting in an overall detection ranging from 8-13% [11,29]. Retrospective studies in fetuses with multiple malformations also obtained a detection rate of causative imbalances from 8 to 15%, including common aneuploidies, by using array [7,30-32]. Our cohort tested by array, however, consisted of a heterogeneous group of fetal deaths, excluding a common aneuploidy which could clarify our lower detection rate.

Overall, in 8.9% of the samples a CNV was detected with unknown inheritance, most of which (12/15) smaller than 1 Mb in size. In contrast to our routine prenatal diagnostic array workflow, in which it is highly recommended to include parental samples simultaneously, in IUFD genetic analysis, testing of parental samples is requested in our written report only after a likely pathogenic CNV has been detected. To reduce the number of results with CNVs of unknown inheritance and therefore unknown clinical relevance, the maximum effort possible should be undertaken to obtain parental blood for testing. Subsequently, finding the cause of the IUFD is helpful to better understand the cause of death, to more accurately determine the recurrence risks, and to enable possible future testing in some cases.

In eight (4.8%) samples a CNV inherited from a healthy parent was detected. Although not all inherited CNVs can always be classified as benign and without clinical relevance with certainty, they are, dependent on the size and the type of CNV, less likely to directly lead to a clinical phenotype: small CNVs (< 0.1 Mb) and gains are less likely to be pathogenic than large CNVs (>1 Mb) and losses, respectively [33,34]. All inherited CNVs in this study were gains ranging in size from ~250 - 1,200 kb, mostly maternally inherited and most likely benign.

The utility of high density SNP arrays is not only useful to rule out potential sample mismatch or false-paternity, but is also used in our laboratory for the examination of regions of homozygosity (ROH) [16,35] and to identify excessive homozygosity, and with specific clinical information the possibility for follow-up diagnostic testing. A nice example in our cohort for demonstrating the power of homozygosity mapping was case id. 336, suspect for Meckel-Grüber syndrome. A homozygous mutation was found in *CEP290*, one of the genes within a large, 80 Mb ROH. Both parents appeared to be heterozygous carriers of this mutation. The Genomic Oligoarray and SNP array evaluation tool enables to evaluate homozygous stretches for autosomal recessive genes in combination with a clinical phenotype making it possible to strategize more focused diagnostic testing [24]. Therefore, we emphasize the need to have detailed phenotypic information to make optimal use of the genotype data from SNP arrays in finding candidate recessive disease genes that may be related to the fetal phenotype.

In concordance with the results of a study of 532 stillbirth samples [36], we conclude that array analysis is more likely to provide an accurate genetic diagnosis than by traditional karyotyping, primarily because of its success with nonviable tissue, making array particularly valuable in the analysis of stillbirths with congenital anomalies. An increased detection rate of chromosomal abnormalities is found when array analysis is used to examine products of conception [37], however, further work is required before the absolute detection rate can be answered.

Nowadays, next-generation sequencing (NGS) is increasingly being used in genetic diagnostics for studying congenital malformations, intellectual disability, and other heterogeneous disorders. Although mutations in genes are known to cause fetal death [38], a systematic genome-wide study has not been performed yet, leaving a large proportion of fetal demise unexplained. It is already demonstrated that NGS not only enables the detection of Single Nucleotide Variation but CNVs as well [39], thus very soon replacing genome wide array analysis in diagnostics. Implementation of NGS for CNV detection and genome/exome wide sequencing in the work-up of IUFD will not only lead to a further improvement of the detection rate, but also to a better fundamental insight in fetal and placental development, and possible maternal interactions.

## Competing interest

The authors declare that they have no competing interests.

## Authors’ contributions

All authors contributed to the interpretation of the diagnostic test findings and participated in the diagnostic service of the Department of Human Genetics. AK compiled the data and wrote most of the manuscript with active cooperation by all authors. All authors read and approved the final version of the manuscript.
